# No association between polymorphisms of *WNT2 *and schizophrenia in a Korean population

**DOI:** 10.1186/1471-2350-11-78

**Published:** 2010-05-24

**Authors:** Hak-Jae Kim, Jin Kyung Park, Su Kang Kim, Sung Wook Kang, Jong Woo Kim, Hyun-Kyung Park, Ah-Rang Cho, Ji Young Song, Joo-Ho Chung

**Affiliations:** 1 College of Medicine, Soonchunhyang University, Chunan 336-745, Republic of Korea; 2 Department of Neuropsychiatry, School of Medicine, Kyung Hee University, Seoul 130-701, Republic of Korea; 3 Kohwang Medical Research Institute, School of Medicine, Kyung Hee University, Seoul 130-701, Republic of Korea; 4 Department of Emergency Medicine, East-West Neo-media Center, Kyung Hee University, 149 Sang-il dong, Gangdong-gu, Seoul 134-727, Republic of Korea

## Abstract

**Background:**

Wingless-type MMTV integration site family member 2 (WNT2) has a potentially important role in neuronal development; however, there has yet to be an investigation into the association between single nucleotide polymorphisms (SNPs) of *WNT2 *and schizophrenia. This study aimed to determine whether certain SNPs of *WNT2 *were associated with schizophrenia in a Korean population.

**Methods:**

e genotyped 7 selected SNPs in the *WNT2 *gene region (approximately 46 Kb) using direct sequencing in 288 patients with schizophrenia and 305 healthy controls.

**Results:**

Of the SNPs examined, one SNP showed a weak association with schizophrenia (p = 0.017 in the recessive model). However, this association did not remain statistically significant after Bonferroni correction.

**Conclusion:**

The present study does not support a major role for *WNT2 *in schizophrenia. This could be due to the size of the population. Therefore, additional studies would be needed to definitively rule out the gene's minor effects.

## Background

Schizophrenia is a complex disease that develops in approximately 0.5-1.0% of the global population and inflicts physical suffering and mental anguish on the affected individuals and their families [1,2]. The neurodevelopmental theory of schizophrenia postulates that abnormal neurodevelopment is one of the etiological factors of schizophrenia [3-6]. Recent studies suggest that schizophrenia may be a subtle disorder of brain development or plasticity [7-9].

Wnt proteins belong to a family of molecules that locally activate cell signaling pathways, regulating cell fate and playing an important role in development [10]. Evidence suggests that Wnt signaling and insulin signaling, which regulates glycogen synthase kinase-3 (GSK-3), may be important in schizophrenia [11]. Several lines of evidence support the association between signaling aberrations and schizophrenia. However, research has shown changes in gene expressions in schizophrenia [12-16], and, in particular, that some such changes affect GSK-3 [14,17-19]. Researchers have also found Wnt signaling abnormalities associated with schizophrenia. For example, expressions of β-catenin proteins decreased in the brains of schizophrenia patients, presumably due to aberrant regulation of β-catenin degradation mediated by activated GSK-3β [20,21]. Another report suggested the enhanced GSK-3β activity was secondary to diminished phosphorylation by Akt, since there is decreased Akt expression in schizophrenia patients' brains [22]. Furthermore, researchers found increased Wnt-1 expression in the hippocampi of schizophrenia patients relative to normal subjects. Wnt-1 is an important molecule in the Wnt pathway [23]. Several studies have implicated Wnt signaling genes in bipolar disorder, as well as in schizophrenia. Researcher found that the frizzled-3 gene (FZD3), which encodes a receptor for Wnt ligands, was associated with schizophrenia in 3 different samples [24-26].

The wingless-type MMTV integration site family, member 2 (*WNT2*) gene is located at chromosome 7q31.2, a region that, in a genome scan study, showed evidence of a link to schizophrenia [27]. *WNT2 *is one of the *WNT *genes that are expressed in a variety of tissues during development [28]. Knockout and expression studies in mice, zebra fish, and *Xenopus *have demonstrated *WNT *genes' crucial roles in the development and patterning of the central nervous system [29-31]. WNT genes encode several signaling proteins that are essential in embryo patterning, cell proliferation, and cell determination [32,33]. The WNT signaling pathway also includes several receptors expressed in both developing and mature nervous systems [34].

Previously, several studies have addressed whether the *WNT2 *gene is associated with autism, a neurodevelopmental disorder [35-37]. Although Wassink et al. reported that one of *WNT2 *SNPs (rs2024233 on exon 5) is associated with autism [35], following association studies did not find any significant associations with autism [36,37]. In addition, Proitsi et al. performed a combined positional and candidate association screen, by identifying known *WNT *signaling genes, and showed that Dickkopf-4 was associated with schizophrenia [38]. There have been no published investigations of the association between *WNT2 *genetic polymorphisms and schizophrenia, despite its potentially important developmental roles and its importance in genetics. In this study, therefore, we investigated the association between WNT2 polymorphisms and schizophrenia in a Korean population.

## Methods

### Subjects

For this study, we evaluated 288 schizophrenic patients (193 males, aged 42.98 ± 10.63 years [mean ± SD]; 95 females, aged 43.03 ± 10.65) and 305 controls with no clinical evidence of any disorders (147 males, 39.92 ± 5.82; 158 females, 36.55 ± 6.77). The schizophrenic patients met the Diagnostic and Statistical Manual of Mental Disorders, Fourth Edition (DSM-IV) [39] criteria for schizophrenia. We reviewed each patient's medical record and rated the schizophrenic patients using the Brief Psychiatric Rating Scale (BPRS) [40], the Scale for the Assessment of Negative Symptoms (SANS) [41], and the operational criteria (OPCRIT) checklist [42].

To evaluate patients for schizophrenia with poor concentration, we used the OPCRIT Checklist. Of the many OPCRIT items, we selected only a few items, already described in our previous study, for use [43]. We determined which patients to place in the poor concentration subgroup according to patients' subjective complaints of being unable to think clearly or make decisions (scoring: 1 for a duration of one week, 2 for two weeks, and 3 for one month) [42]. Thus, we examined 167 schizophrenic patients having poor concentration (116 males, 42.8 ± 10.0; 51 females, 41.9 ± 10.8) and 121 schizophrenic patients who had no such complaints (77 males, 43.6 ± 11.2; 44 females, 44.8 ± 10.7). We also analyzed the associations between the schizophrenic patients' SNPs and their SANS scores (Table 1). The genotype frequencies of rs6948009 was evaluated (A/A, n = 211; A/G, n = 71; G/G, n = 3) for the analysis with SANS scores.

**Table 1:** *WNT2 *SNPs genotype and allele frequencies in schizophrenia patients and healthy controls

		Schizophrenia		Control				
					
SNP	Genotype	Freq	%	Freq	%	Model	OR (95% CI)	P value
rs39315	A/A	90	0.31	79	0.26	co-dominant	1.21 (0.95-1.54)	0.12
5'-near gene	A/G	137	0.48	150	0.49	dominant	1.38 (0.94-2.02)	0.1
	G/G	61	0.21	76	0.25	recessive	1.20 (0.80-1.81)	0.38

rs17132543	A/A	97	0.34	98	0.32	co-dominant	1.14 (0.89-1.45)	0.3
Intron4	A/G	138	0.48	145	0.48	dominant	1.16 (0.80-1.68)	0.43
	G/G	53	0.18	62	0.2	recessive	1.22 (0.79-1.89)	0.37

rs3779548	A/A	127	0.44	135	0.44	co-dominant	0.97 (0.74-1.25)	0.8
Intron4	A/G	128	0.44	136	0.45	dominant	0.99 (0.70-1.40)	0.96
	G/G	33	0.11	34	0.11	recessive	0.88 (0.50-1.53)	0.65

rs733154	A/A	171	0.59	177	0.58	co-dominant	1.00 (0.74-1.33)	0.97
Intron4	A/G	103	0.36	109	0.36	dominant	1.02 (0.72-1.44)	0.93
	G/G	14	0.05	19	0.06	recessive	0.89 (0.42-1.92)	0.78

rs2024233	A/A	87	0.3	84	0.28	co-dominant	1.11 (0.87-1.43)	0.41
3'-UTR	A/G	148	0.51	157	0.51	dominant	1.15 (0.79-1.69)	0.46
	G/G	53	0.18	64	0.21	recessive	1.14 (0.74-1.77)	0.54

rs4730775	G/G	176	0.61	184	0.6	co-dominant	0.89 (0.66-1.20)	0.45
3'-near gene	G/A	94	0.33	112	0.37	dominant	1.02 (0.71-1.45)	0.93
	A/A	17	0.06	9	0.03	recessive	0.35 (0.14-0.85)	**0.017**

Freq, frequency; OR, odds ratio; CI, confidence interval.

We recruited control subjects who had been found mentally fit by a general health checkup program. All studies were carried out according to the guidelines of the Declaration of Helsinki [44]. We obtained written informed consent from each subject. The study was approved by the ethics review committee of the Medical Research Institute, Kyung Hee University Medical Center, Seoul, Republic of Korea.

### SNP Genotyping

**Figure 1:**
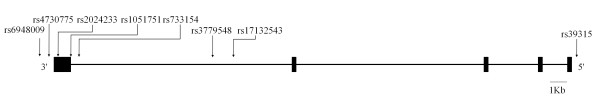
**Gene map and single nucleotide polymorphisms (SNPs) in the *WNT2 *gene on chromosome 7q31.2**. Exons are marked with boxes. The coding regions are black boxes. The first nucleotide is denoted as +1. The arrow indicates the location of each SNP.

We selected 8 SNPs from *WNT2 *genes in chromosome 7q31.2 by downloading all the SNPs typed in the *WNT2 *genes from the HapMap database (http://www.hapmap.org/;genome build 35) and dbSNP database version 129. From these, we selected tag SNPs using the Tagger program's aggressive tagging option (Paul de Bakker, http://www.broad.mit.edu/mpg/tagger/), so as to capture SNPs with a minor allele frequency of < 5% (*r*^2 ^> 0.8). This gave 2 exonic SNPs (rs2024233 and rs1051751), 3 intronic SNPs (rs733154, rs3779548, and rs17132543), and 3 regulatory SNPs (thought to be the promoter; the intervals are 2000 bp up- and downstream between the genes) (rs6948009 [869 bp downstream from the last nucleotide of the 3'-UTR], rs4730775 [164 bp downstream from the last nucleotide of the 3'-UTR], and rs39315 [219 bp upstream from the first nucleotide of the 5'-UTR]). In addition, we chose these SNPs because they were previously evaluated for relation to schizophrenia risk or showed evidence of functional significance. Figure 1 shows the locations of the selected SNPs.

Using a commercially available Qiagen DNA Extraction kit (Qiagen, Tokyo, Japan), we extracted genomic DNA from blood samples that had been placed in EDTA. We amplified the genomic DNA using the primers shown in Table 2 for each SNP, sequenced the PCR products using an ABI Prism 377 automatic sequencer (PE Applied Biosystems, Foster City, California, USA), and analyzed the sequence data using SeqManII software (DNASTAR Inc., Madison, Wisconsin, USA).

**Table 2:** Primer Sequences

SNP	Sequence(5'-3')	Product Size	Temperature
rs39315	Forward CCTCCCTATGGGCTCTGTATT	450	60
	Reverse CACGGGTGCATGAAATGATGG		

rs17132543	Forward AGCCTCTAGAGAAGTCCTGAAG	373	60
	Reverse CTCCCAACCACACTCACACACA		

rs3779548	Forward GTGTGGCCTACTTTGCAGAAG	355	60
	Reverse TTCTCCAGCACCTAGACTGTG		

rs733154	Forward GGATCCTTGATCGAGCAGAGCCA	301	60
	Reverse GACTGCAGCAGGAGAGACAGTTA		

rs1051751	Forward TGGGCCCACAGAACGAGTATAAC	327	65
	Reverse CCAGAGCTTCCAGGCAGTCCT		

rs2024233	Forward GTAACAAGGTGGGGACGTGTGT	319	62
	Reverse GAGATTCCATGGGTCACATGCA		

rs4730775	Forward TGGGGATACAAGATTGGTGAAC	360	65
	Reverse GATGGCAGAAGCCAACCACTA		

rs6948009	Forward GGTCATTTAGACTGAGACTCG	461	60
	Reverse CACCAATCCCTTCGCCTCTCT		

### Statistical analysis

Using SNPstats, we assessed the Hardy-Weinberg equilibrium (HWE) for both controls and schizophrenia patients [45]. For the linkage disequilibrium (LD) block, we used the Haploview version 3.32 [46]. We inferred the haplotypes and their frequencies using the EM algorithm [47]. We used the multiple logistic regression model to calculate odds ratios (OR), 95% confidence intervals, and corresponding *p *values (controlling age and gender as covariables), to analyze the association between schizophrenia and both SNPs and haplotypes. Furthermore, we analyzed the association between the schizophrenia subgroup and both SNPs and haplotypes, using SNPstats, the HapAnalyzer version 1.0 [48], and Helixtree (Golden Helix Inc., MT, USA). We assessed the associations between SNPs and SANS scores, using one-way ANOVA to analyze the relationship between SANS scores and genotype frequencies in the schizophrenia subgroup. We calculated the power, given the sample size, using a genetic power calculator http://pngu.mgh.harvard.edu/~purcell/gpc[49], and, to reduce error, we adjusted the effective sample size (calculated sample size × 100/95). When we calculated the sample powers, we found our case-control study was sufficiently powerful to determine a positive association. In this study, we found sample powers of 0.8046 (rs39315, effective sample size, number of cases for 80% power = 284), 0.8493 (rs17132543, n = 252), 0.9338 (rs3779548, n = 188), 0.9677 (rs733154, n = 155), 0.9145 (rs2024233, n = 208), and 0.9689 (rs4730775, n = 154) for detecting a two-fold increased risk, assuming an *α*-level of 0.05. However, the sample powers in the divided schizophrenia subgroup (with or without poor concentration) were not sufficient (data not shown). We applied the Bonferroni correction by multiplying *P *values by the number of SNPs analyzed (*n *= 6).

## Results

Of the eight SNPs we examined, all were polymorphic. The genotype distributions of six SNPs (rs39315, rs17132543, rs3779548, rs733154, rs2024233, and rs4730775) were in HWE (*P *> 0.05). We did not estimate one SNP (rs6948009) for the association analysis because its genotype distribution was not in HWE (*p *< 0.05). Also, we excluded one SNP (rs1052751) from the analysis, since it was not polymorphic. Of these SNPs, one SNP (rs4730775) was weakly associated with schizophrenia. Despite this association (p = 0.017 in the recessive model), we could not prove the statistical significance of analysis after the Bonferroni correction. Thus, these results do not support a significant role for the *WNT2 *sequence variation in the etiology of schizophrenia.

**Figure 2:**
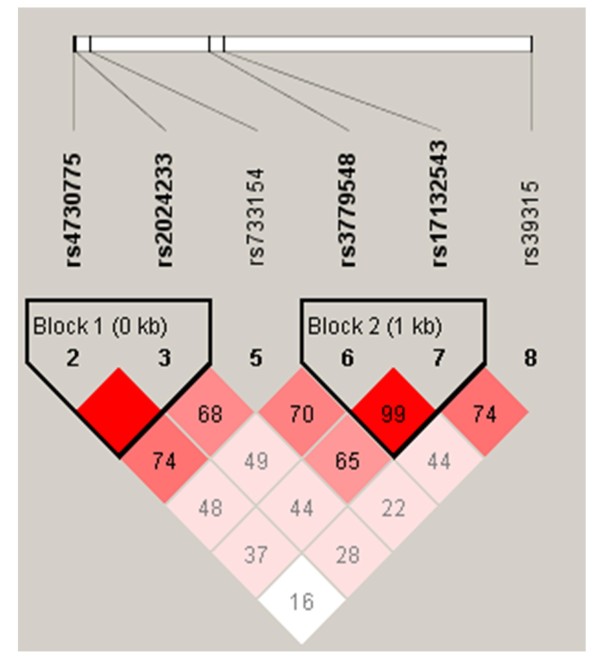
**Linkage disequilibrium (LD) blocks of the *WNT2 *gene**. LD coefficient (|D'|) and LD blocks among *WNT2 *SNPs. Block 1 consists of rs4730775 and rs2024233. Block 2 comprises rs3779548 and rs17132543.

To further analyze the haplotype structure in our samples, we characterized the two LD blocks between the six *WNT2 *SNPs (including the 5'UTR and 3'UTR) in the control subjects, using the pairwise D'values (Figure 2). Per the criteria used by Gabriel et al. [50], the LD data revealed two haplotype blocks across *WNT2*. The D' values from rs4730775 to rs32024233 and from rs37795488 to rs17132543 ranged between 0.95 and 0.99, indicating strong LD between each pair of markers (Figure 2). We performed a haplotype-based association analysis between the schizophrenic and control groups for different combinations of SNPs within *WNT2*, revealing two LD blocks (block 1, rs4730775 and rs32024233; block 2, rs37795488 and rs17132543). However, we did not detect significant associations between the haplotypes in block 1 or 2 and schizophrenia (all p-values of the analysis > 0.05; Table 3). We then assessed the gene and haplotype associations with these SNPs in the poor-concentration schizophrenia subgroup (determined using the OPCRIT Checklist). In this subgroup, the genotype distributions of the seven SNPs considered were in HWE (*p *> 0.05). We then analyzed the genetic associations of these SNPs within the subgroup. The SNPs of the *WNT2 *gene were not associated with poor concentration, a clinical symptom of schizophrenia (Table 4). In addition, we assessed the associations between SNPs and SANS scores. No statistically significant differences appeared in the total SANS scores among the seven SNPs (Table 5). To compare our genotypic results with different ethnic populations, we searched the human SNP database (http://www.ncbi.nlm.nih.gov/SNP dbSNP Build 130). This database presents genotype frequencies for the SNPs analyzed in this study (Table 6). The control group's genotype distributions of the SNPs we analyzed are similar to those of Asian populations, especially the Japanese population (Table 6).

**Table 3:** *WNT2 *SNPs haplotype frequencies in schizophrenia patients and healthy controls.

Haplotype		Schizophrenia			Control					
(Block 1)	Type	Freq	%	Freq	Freq	%	Freq	Models	OR(95% CI)	P value
	HH	51	0.19		66	0.21		co-dominant	0.92(0.73-1.16)	0.4778
HAP1 GG	H-	142	0.52	0.44	163	0.51	0.46	dominant	0.91(0.64-1.31)	0.6236
	--	82	0.3		89	0.28		recessive	0.87(0.58-1.31)	0.5004

	HH	31	0.11		37	0.12		co-dominant	1.04(0.82-1.32)	0.7506
HAP2 GA	H-	121	0.44	0.33	132	0.42	0.32	dominant	1.09(0.79-1.51)	0.604
	--	123	0.45		149	0.47		recessive	0.96(0.58-1.60)	0.8901

	HH	15	0.05		11	0.03		co-dominant	1.07(0.81-1.42)	0.6325
HAP3 AA	H-	93	0.34	0.22	113	0.36	0.21	dominant	1.01(0.73-1.41)	0.9446
	--	167	0.61		194	0.61		recessive	1.61(0.73-3.57)	0.2405

										

**Haplotype**		**Schizophrenia**			**Control**					
**(Block 2)**	**Type**	**Freq**	**%**	**Freq**	**Freq**	**%**	**Freq**	**Models**	**OR(95% CI)**	**P value**

	HH	50	0.18		65	0.2		co-dominant	0.90(0.72-1.14)	0.3894
HAP1 AG	H-	130	0.47	0.42	152	0.48	0.44	dominant	0.88(0.63-1.24)	0.4723
	--	95	0.35		101	0.32		recessive	0.86(0.57-1.30)	0.4881

	HH	31	0.11		35	0.11		co-dominant	1.01(0.80-1.29)	0.912
HAP2 GA	H-	123	0.45	0.34	142	0.45	0.33	dominant	1.01(0.73-1.40)	0.9338
	--	121	0.44		141	0.44		recessive	1.03(0.62-1.72)	0.9181

	HH	15	0.05		14	0.04		co-dominant	1.12(0.86-1.48)	0.4011
HAP3 AA	H-	104	0.38	0.24	114	0.36	0.22	dominant	1.13(0.82-1.57)	0.4568
	--	156	0.57		190	0.6		recessive	1.25(0.59-2.64)	0.5543

Freq, frequency; OR, odds ratio; CI, confidence intervals.

**Table 4:** *WNT2 *genotype frequencies in Korean schizophrenia patients with poor concentration

		NPC		PC				
					
SNP	Genotype	Freq	%	Freq	%	Model	OR (95% CI)	P value
rs39315	A/A	40	33.1	50	29.9	co-dominant	0.97 (0.70-1.34)	0.85
5'-near gene	A/G	52	43.0	85	50.9	dominant	1.16 (0.70-1.92)	0.57
	G/G	29	24.0	32	19.2	recessive	0.75 (0.43-1.33)	0.33

rs17132543	A/A	40	33.1	57	34.1	co-dominant	0.99 (0.71-1.38)	0.95
Intron4	A/G	59	48.8	79	47.3	dominant	0.96 (0.59-1.58)	0.88
	G/G	22	18.2	31	18.6	recessive	1.02 (0.56-1.87)	0.95

rs3779548	A/A	50	41.3	77	46.1	co-dominant	0.90 (0.63-1.27)	0.54
Intron4	A/G	57	47.1	71	42.5	dominant	0.82 (0.51-1.32)	0.42
	G/G	14	11.6	19	11.4	recessive	0.99 (0.47-2.06)	0.97

rs733154	A/A	67	55.4	104	62.3	co-dominant	0.84 (0.56-1.27)	0.41
Intron4	A/G	50	41.3	53	31.7	dominant	0.71 (0.44-1.15)	0.17
	G/G	4	3.3	10	6.0	recessive	1.77 (0.53-5.89)	0.34

rs2024233	A/A	38	31.4	49	29.3	co-dominant	1.11 (0.79-1.57)	0.53
3'-UTR	A/G	63	52.1	85	50.9	dominant	1.10 (0.66-1.83)	0.72
	G/G	20	16.5	33	19.8	recessive	1.23 (0.67-2.28)	0.50

rs4730775	G/G	70	57.9	106	63.9	co-dominant	0.87 (0.59-1.28)	0.48
3'-UTR	G/A	45	37.2	49	29.5	dominant	0.76 (0.47-1.24)	0.27
	A/A	6	5.0	11	6.6	recessive	1.29 (0.46-3.62)	0.62

rs6948009	G/G	87	73.1	124	74.7	co-dominant	0.80 (0.48-1.33)	0.39
3'-near gene	G/A	29	24.4	42	25.3	dominant	0.89 (0.52-1.53)	0.67
	A/A	3	2.5	0	0	recessive	0.00 (0.00-NA)	0.02

Freq, frequency; NPC, nonpoor concentration; PC, poor concentration; OR, odds ratio; CI, confidence interval; N/A, not applicable.

**Table 5:** Comparison of SANS scores between schizophrenic patient groups with different genotypes of *WNT2*.

			SANS		
			
SNP	Genotype	n	Mean	S.E.	P value
rs39315	A/A	90	66.78	2.627	0.388
5'-near gene	A/G	137	63.76	2.239	0.866
	G/G	61	64.41	2.831	0.55

rs17132543	A/A	97	63.99	2.46	0.458
Intron4	A/G	138	66.51	2.256	0.278
	G/G	53	62.06	3.016	0.63

rs3779548	A/A	127	66.42	2.139	0.689
Intron4	A/G	128	65.17	2.257	0.124
	G/G	33	57.48	4.394	0.062

rs733154	A/A	171	66.11	1.946	0.371
Intron4	A/G	103	63.32	2.375	0.684
	G/G	14	60.5	6.791	0.429

rs2024233	A/A	87	60.26	2.717	0.031
3'-UTR	A/G	148	67.59	2.034	0.461
	G/G	53	64.68	3.319	0.311

rs4730775	G/G	176	65.82	1.85	0.267
3'-UTR	G/A	94	64.23	2.63	0.619
	A/A	17	58.82	6.56	0.427

rs6948009	A/A	211	65.02	1.81	0.981
3'-near gene	A/G	71	64.94	2.5	0.307
	G/G	3	52.33	3.38	0.406

SANS, Scale for the Assessment of Negative Symptoms; S.E., Standard Error; SNP, single nucleotide polymorphism.

**Table 6:** *WNT2 *SNPs genotype frequencies in each population

SNP	Genotype	Schizophrenia	Control	Europe	China	Japan	Sub-Saharan African
rs39315	A/A	0.31	0.26	0.36	0.29	0.46	0.22
5'-near gene	A/G	0.48	0.49	0.41	0.62	0.43	0.44
	G/G	0.21	0.25	0.22	0.09	0.11	0.34

P value				0.285	0.010	0.003	0.373

rs17132543	A/A	0.34	0.32	0.78	0.24	0.44	0.97
Iintron4	A/G	0.48	0.48	0.22	0.58	0.47	0.03
	G/G	0.18	0.20	0.00	0.18	0.09	0.00

P value				0.000	0.334	0.048	0.000

rs3779548	A/A	0.44	0.44	0.30	0.69	0.36	0.17
Intron4	A/G	0.44	0.45	0.47	0.22	0.44	0.65
	G/G	0.11	0.11	0.23	0.09	0.20	0.18

P value				0.031	0.001	0.180	0.000

rs733154	A/A	0.59	0.58		0.85	0.62	0.31
Intron4	A/G	0.36	0.36	No data	0.13	0.31	0.37
	G/G	0.05	0.06		0.03	0.08	0.33

P value				N/A	0.000	0.675	0.000

rs1051751	G/G	1.00	1.00	1.00	1.00	0.98	1.00
Exon 5	G/T	0.00	0.00	0.00	0.00	0.02	0.00
Cys294Phe	T/T	0.00	0.00	0.00	0.00	0.00	0.00

P value				N/A	N/A	N/A	N/A

rs2024233	A/A	0.30	0.28	0.50	0.29	0.34	0.54
3'-UTR	A/G	0.51	0.51	0.40	0.44	0.50	0.41
	G/G	0.18	0.21	0.10	0.27	0.16	0.05

P value				0.003	0.526	0.531	0.000

rs4730775	G/G	0.61	0.60	0.37	0.71	0.62	0.46
3'-near gene	G/A	0.33	0.37	0.36	0.27	0.29	0.45
	A/A	0.06	0.03	0.27	0.02	0.09	0.09

P value				0.000	0.261	0.135	0.060

rs6948009	A/A	0.74	0.53		0.95	0.93	
3'-near gene	A/G	0.25	0.45	No data	0.05	0.05	No data
	G/G	0.01	0.02		0.00	0.02	

P value				N/A	0.000	0.000	N/A

From database http://www.ncbi.nlm.nih.gov/sites/entrez?db=snp. We estimated all p-values via comparisons between the control group of our sample and each population. N/A, not applicable.

## Discussion

The *WNT2 *gene and its chromosomal location have received attention as a candidate gene with regard to autism. Given the similarities between autistic disorders and schizophrenia (as a neurodevelopmental disorder), we investigated whether *WNT2 *gene variations act as risk factors for schizophrenia in a Korean sample. The results suggest that *WNT2 *may not be involved in the pathogenesis of schizophrenia. Of all the SNPs and haplotypes analyzed, only one SNP (rs4730775) showed a weak association with the disorder. However, the significance disappeared after the Bonferroni correction for multiple testing (p = 0.102, Table 1). In addition, we investigated the LD between the *WNT2 *SNPs and performed a haplotype analysis between the schizophrenia and control subjects. We could not find any association between *WNT2 *and schizophrenia, indicating that there is no genetic association between *WNT2 *and schizophrenia.

To further analyze the association test between clinical symptoms and SNP genotypes, we evaluated other symptoms, from the OPCRIT checklist and the total SANS scores. However, we found no statistically significant association between any symptom in OPCRIT (Table 4) and total SANS score (Table 5). We compared our genotype frequencies with the human SNP database http://www.ncbi.nlm.nih.gov/sites/entrez?db=snp, to show ethnic similarities and differences. The genotype frequencies of our study sample resemble those of the Japanese and Chinese Hapmap populations (Table 6). Further studies are necessary to elucidate (i) whether another case-control study is appropriate and (ii) whether promoter SNPs can affect the expression of *WNT2*. To confirm or refute the lack of association between the *WNT2 *gene and schizophrenia, replication studies with adequate sample sizes, or studies with additional SNPs not analyzed in the present study, may be required.

This report has several limitations. First, the sample size may not have been sufficient to detect associations of smaller effects on schizophrenia. We estimated that this sample had more than 0.8 powers to detect association, with the gene exerting a genotypic relative risk of approximately 2. However, schizophrenia appears to be a very genetically complex disease, and the *WNT2 *gene risks may be smaller than 2. Second, when we designed the experiment, the versions of the HapMap database and dbSNP database were slightly different compared to their present forms, and, as a result, our selection of SNPs provided incomplete coverage of currently-known common variations in the *WNT2 *gene. Furthermore, the present databases showed different locations for some SNP from previous databases. Consequently, we may have missed some of the relevant associations of *WNT2 *genes.

## Conclusions

We investigated possible associations between *WNT2 *gene SNPs and schizophrenia, and the results were negative. For that reason, it appears the SNPs of *WNT2 *may not influence the development of schizophrenia in the Korean population, but, still, additional genetic studies will help develop understanding of the precise mechanisms underlying pathogenesis in schizophrenic patients.

## Competing interests

The authors declare that they have no competing interests.

## Authors' contributions

Authors JWK and JHC designed and directed the whole project. Authors SKK and SW K managed the literature searches and analyses. JYS, ARC, and JKP carried out the schizophrenia assessments and advised on patient selection. Author HKP advised on control group collection. Author HJK performed most of the statistical analyses and the genotyping and contributed substantially to the first draft of the manuscript. All authors contributed to and have approved the final manuscript

## Pre-publication history

The pre-publication history for this paper can be accessed here:

http://www.biomedcentral.com/1471-2350/11/78/prepub
